# Post Typhoid Sepsis

**Published:** 1903-10-03

**Authors:** 


### POST-TYPHOID SEPSIS.

Under this heading, Dr. Delafield[1] discusses those cases where, after an attack of typhoid, a patient has fever which cannot be attributed to a relapse. He points out the importance of making a distinction between what may be considered as a complication of enteric fever and a relapse which is really a fresh attack of typhoid. Thus there may be inflammation of the gall-bladder, of the urinary bladder, of connective tissue—all due to infection with typhoid baccilli, but they are not typhoid fever. In the same way, after typhoid fever there are fevers which may be due to typhoid baccilli or to other infections, but they are not typhoid fever. Among these may be distinguished (1) the ordinary moderate daily rises of temperature, only lasting for a few hours, occurring within three weeks after the end of an attack of typhoid fever. These rises of temperature would probably be overlooked entirely unless the thermometer were systematically used, for they are accompanied by no symptoms and do no harm. The only necessary thing is to know that they are unimportant. (2) The post-typhoid fevers which are of real importance, and last for one or more weeks, but yet do not make the patient very ill and are not fatal. This form of fever occurs quite frequently. The rise of temperature may be continuous with the typhoid temperature, or there may be an interval of several days of normal temperature before the post-typhoid fever begins. The temperature is of remittent type, the rises sometimes being as high as 105° F. With this fever there is no delirium or sleeplessness, the tongue is moist and clean, there is no eruption, no tympanites, no diarrhœa. The patients are hungry, and if kept on low diet they may have rather alarming attacks of heart failure. As long as they are kept in bed and on a fluid diet there is no particular limit to the duration of the fever. The proper treatment is to get them out of bed and feed them up, when they rapidly recover strength and put on flesh. (3) More important than these mild fevers are the severe and long-continued post-typhoid fevers which may terminate fatally. They occur as a rule after severe cases of typhoid fever and the septic fever is continuous with the typhoid fever. The temperature is remittent, rising to 102° or 103° in the afternoon, with perhaps occasional sudden jumps to 105° or 106°. The patient looks septic rather than in the typhoid state. The mind is often dull and apathetic, but there is no active delirium. The tongue is dry, there may be nausea and vomiting, and yet there is some desire for food. There is a rapid loss of flesh and strength so that the condition may become alarming. Thrombosis of one or both femoral veins may occur. If the patients are insufficiently fed they are very apt to die. In the autopsies of the fatal cases which he has made, Dr. Delafield has found the intestinal lesions healed, while no cause of death was apparent beyond sepsis and starvation.

In these cases, as in the milder forms, the correct treatment is to get the patients out of bed and give them plenty of food. Then they gain flesh and strength even while the fever continues. Such treatment will call for a good deal of courage on the part of the medical man in attendance on the patient, but Dr. Delafield is quite convinced as to the correctness and success of the method he suggests.

[1] Medical Record.

